# Unilateral Leg Edema and Hydronephrosis in IgG4-Related Retroperitoneal Fibrosis

**DOI:** 10.31662/jmaj.2023-0057

**Published:** 2023-09-27

**Authors:** Takahiro Kobayashi, Naoya Fujita, Akinori Sekizawa, Yosuke Ono

**Affiliations:** 1 Department of General Medicine, National Defense Medical College, Saitama, Japan

**Keywords:** IgG4-related retroperitoneal fibrosis, hydronephrosis, leg edema

A 69-year-old man presented with a 2-month history of left leg edema. A contrast-enhanced computed tomography (CT) scan showed a mass occupying the caudal retroperitoneal area surrounding the pelvic arteries and veins (Figure 1A, red arrow). Laboratory findings demonstrated a high serum IgG4 concentration of 304 mg/dL (reference range, 11-121 mg/dL) greater than 135 mg/dL. Although the leg edema subsequently disappeared, the enlarging mass suppressed the right ureter, causing right-side hydronephrosis (Figure 1B, red arrow). His worsening renal dysfunction warranted ureteral stenting; however, he refused the procedure. A specimen from the retroperitoneal mass obtained using CT-guided biopsy revealed lymphoplasmacytic infiltration with storiform fibrosis, the number of IgG4-positive plasma cells greater than 10 per high powered field, and the ratio of IgG4-positive plasma cells/IgG-positive cells greater than 40% (Figure 1C, 1D and Figure 1E), leading to a definitive diagnosis of IgG4-related disease ^(1), (2)^. After 2 months of oral prednisolone and azathioprine treatment, the mass shrank and hydronephrosis subsequently improved (Figure 1F and 1G, red arrow). This case highlights the importance of evaluating the anatomical compression of veins and ureters by a retroperitoneal mass. Invasive ureteral stenting for hydronephrosis can be avoided if immunosuppressive treatment is effective for IgG4-related retroperitoneal fibrosis. Shared decision-making through patient-centered care was useful even in situations where the progressive organ dysfunction requiring surgical intervention was inevitable.

**Figure 1.**
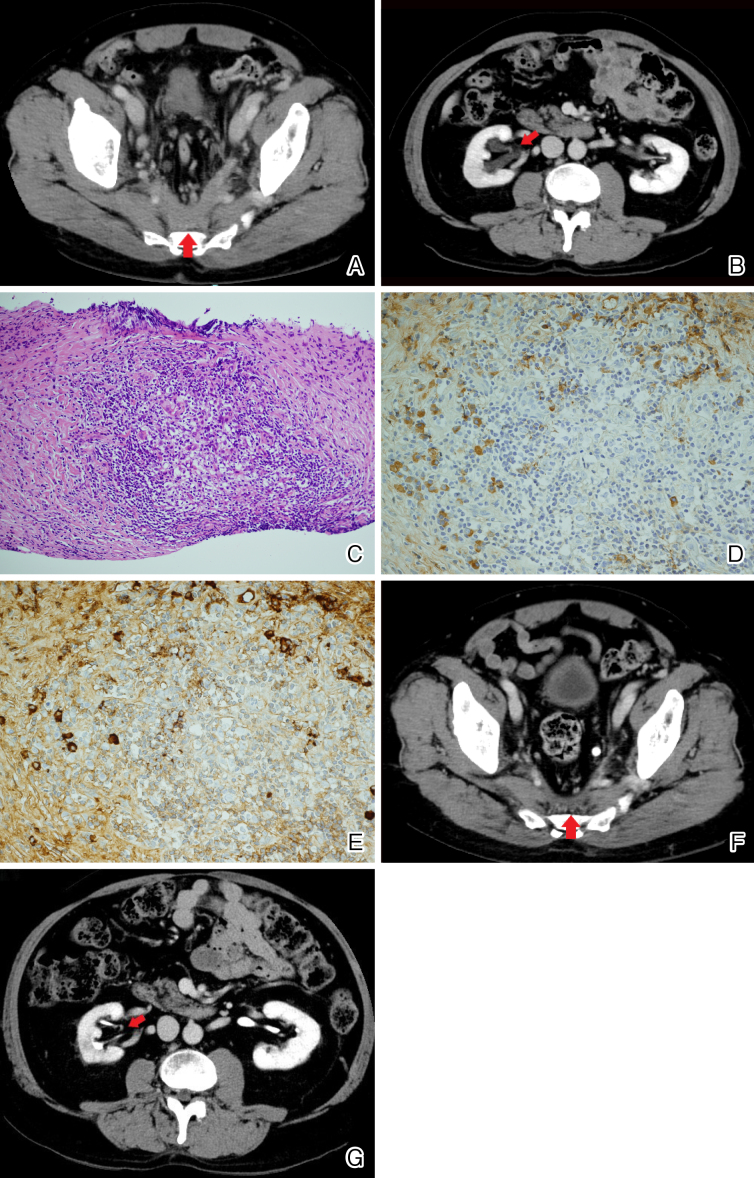
(A, B) Contrast-enhanced computed tomography scans before treatment showing a mass (red arrow) occupying the caudal retroperitoneal area surrounding the pelvic arteries and veins (A) and an enlarging mass that suppressed the right ureter, causing right-side hydronephrosis (red arrow) (B). (C, D, E) Hematoxylin and eosin staining of core needle biopsy specimens taken from retroperitoneal mass showing stromal fibrosis with dense infiltration of normal lymphocytes and plasma cells. (magnification, × 200) (C). Immunohistochemical staining showing the infiltration of IgG-positive plasma cells (magnification, × 400) (D). The number of IgG4-positive plasma cells per high-power field was 30 (magnification, × 400) (E). The ratio of IgG4-positive plasma cells/IgG-positive cells was approximately 60%. (F, G) After 2 months of treatment with oral prednisolone and azathioprine, the mass shrank (red arrow) (F) and the hydronephrosis subsequently improved (red arrow) (G).

## Article Information

### Conflicts of Interest

None

### Sources of Funding

This work was supported by the Japan Medical Education Foundation (https://www.jmef.or.jp/).

### Author Contributions

TK wrote the first draft of the manuscript and NF, AS, and YO revised the manuscript. Furthermore, TK and NF contributed to patient care and YO organized the manuscript.

### Informed Consent

The patient provided written informed consent for the publication of the manuscript and clinical images.

### Approval by Institutional Review Board (IRB)

This study did not require IRB approval.
